# Impact of the Use of Interactive Screens on Language Development in Children up to 6 Years of Age: A Systematic Review

**DOI:** 10.1111/cch.70176

**Published:** 2025-11-14

**Authors:** Djalma Carmo da Silva Junior, Yasmin Marques Castro, Rinelly Pazinato Dutra, Douglas Pinheiro Caumo, Michael Pereira da Silva

**Affiliations:** ^1^ School of Medicine Federal University of Rio Grande Rio Grande Brazil

**Keywords:** audiovisual media, child development, interactive screens, language development, systematic review

## Abstract

**Objective:**

The study aims to examine the association between interactive screen use and language development in children up to 6 years of age through a systematic review of observational studies.

**Methods:**

This systematic review was conducted according to the *Preferred Reporting Items for Systematic Reviews and Meta‐Analyses* (PRISMA) guidelines. The literature search included four databases and used the PECOS strategy. Observational studies—either cross‐sectional or longitudinal—that investigated the relationship between the use of mobile devices (such as smartphones and tablets) and language development in children aged 0–6 years were included. Duplicate records were removed using Rayyan software. Screening was carried out in pairs in two stages: title/abstract review and full‐text reading.

**Results:**

Most of the evidence suggests a negative association between excessive screen time and language development, particularly in expressive language and vocabulary. However, some studies found no statistically significant association, suggesting that factors such as exposure time, quality of interactions and family context influence the observed outcomes. Methodological heterogeneity limited direct comparisons between findings.

**Conclusions:**

Although the results are not consistent, there is evidence of negative effects of prolonged and unsupervised use of electronic devices on young children's language development.

## Summary

- Early and excessive exposure to interactive screens may negatively affect expressive language and vocabulary development in children under 6 years of age.
- The context and quality of screen use—including parental mediation, co‐viewing, and the type of content accessed—are crucial in determining its impact on language development.
- Health professionals and educators should guide families on the importance of supervised, limited and purposeful screen use during early childhood.
- Public health policies should reinforce the dissemination of screen time guidelines and promote media literacy among caregivers to reduce potential developmental risks.

## 1 Introduction

Language development is a complex process that begins in childhood and involves basic skills such as speaking, understanding, reading and writing. This process takes place in stages that reflect cognitive and social changes and is essential for communication and the child's overall development (McKean and Reilly 2023).

In recent years, the use of interactive screen‐based digital devices, such as tablets and smartphones, has become increasingly present in children's lives, raising concerns about its impact on development. In this review, the focus was on interactive screen devices, mainly tablets and smartphones, distinguishing them from passive screen exposure (e.g., television), as their interactive nature may have different implications for language development.

Excessive screen time has been linked to deficits in language, communication, motor skills and socio‐emotional health (Madigan, Browne, et al. 2019). It can also contribute to sedentary lifestyles, obesity, metabolic and cardiovascular disease, impair social interaction, disrupt sleep and expose children to inappropriate content (Krupa et al. 2019).

On the other hand, interactive resources can stimulate curiosity and encourage learning, provided they are used in a balanced and supervised way (Selfa‐Sastre et al. 2022). The challenge is to understand how exposure to these technologies affects children's language development. Studies suggest that excessive time spent in front of screens can reduce social interactions and compromise time spent on activities that are essential for language learning (Alroqi et al. 2023).

Faced with this problem, several countries have introduced regulations on the use of screens to minimize the negative impact on children's development (Amos et al. 2023; Gupta et al. 2022; Straker et al. 2018). The World Health Organization (WHO) recommends that children under the age of 1 should not be exposed to screens and that screen time should be limited to 1 h a day between the ages of 2 and 4 (Tadpatrikar et al. 2024). These guidelines reflect a growing concern about the impact of early exposure to digital technologies on children's development, particularly with regard to language acquisition and social interactions. However, there is still disagreement in the literature about the actual extent of these effects and what factors may mitigate or enhance them. In this context, this study aims to investigate the effects of interactive screen use on language development in children up to 6 years of age through a systematic review of observational studies.

## 2 Method

### 2.1 Design

This systematic review was conducted following the Preferred Reporting Items for Systematic Reviews and Meta‐Analyses (PRISMA) guidelines (Page et al. 2021). The review protocol was prospectively registered on PROSPERO (International Prospective Register of Systematic Reviews) under the registration number CRD42024550692 on 04 June 2024.

### 2.2 Research Question and PECOS Strategy

To conduct the systematic review, the guiding question was formulated using the PECOS strategy (an acronym for [P] patient, [E] exposure, [C] comparison, [O] outcomes and [S] study type, i.e., patient, intervention, comparison, outcomes and study type). According to Santos et al. (2007), this strategy includes the five basic elements for bibliographic searches for evidence and the construction of research in evidence‐based practice. Thus, the question formulated was as follows: “What is the effect of using interactive screens on language development in children up to 6 years of age?”, and the PECOS strategy was consolidated as follows:

- P = children aged 0–6 years;
- E = interactive screens (smartphones and tablets);
- C = no screen use or shorter exposure time;
- O = language development;
- S = observational studies (cross‐sectional and longitudinal).

### 2.3 Databases and Search Strategies

The databases used for the bibliographic search were the Scopus platform, EMBASE, PubMed and the Latin American and Caribbean Literature on Health Sciences (LILACS). The descriptors used to formulate the search strategies were selected from the Health Sciences Descriptors (DeCS) provided by the Virtual Health Library (VHL) and the Medical Subject Headings (MeSH) terms adopted from PubMed.

The main search terms used were as follows:

- **Population:** *Child*, *Children*, *Kids*, *Preschool Child*, *Early Childhood*, *Infant*, *Toddler*, *Preschooler*, and *Paediatrics*
- **Exposure:** *Smartphone*, *Phone*, *Cell*, *Mobile*, *Tablets*, *Screens*, *Interactive Screens*, *Mobile Device* and *Screen Device*
- **Outcomes:** *Language* and *Language Development*

A complementary search was carried out in the grey literature by analysing the first 100 results in Google Scholar, as well as a manual search in the reference lists of reviews and included studies. The complete search strategies for each database are detailed in Table 1.

**TABLE 1:** Search strategies.

Databases	Search strategies
MedLine via PubMed	((“children”[All Fields] OR “kids”[All Fields] OR “early childhood”[All Fields] OR “toddler”[All Fields] OR “preschooler”[All Fields] OR (“Child”[MeSH Terms] OR “child, preschool”[MeSH Terms] OR “Infant”[MeSH Terms] OR “Paediatrics”[MeSH Terms])) AND (“smartphone”[MeSH Terms] OR “phone”[All Fields] OR “cell”[All Fields] OR “mobile”[All Fields] OR “tablets”[MeSH Terms] OR “screens”[All Fields] OR “interactive screens”[All Fields] OR “mobile device”[All Fields] OR “screen device”[All Fields]) AND (“language”[MeSH Terms] OR “language development”[MeSH Terms])) AND ((ffrft[Filter]) AND (2010:2024[pdat]))
Lilacs	(child) OR (child, preschool) OR (infant) OR (paediatrics) AND (smartphone) OR (tablets) AND (language) OR (language development)
Embase	((child:ti,ab,kw OR (kid:ti,ab,kw AND goat:ti,ab,kw) OR ‘early childhood’:ti,ab,kw OR toddler:ti,ab,kw OR preschoolers:ti,ab,kw OR ‘preschool child’:ti,ab,kw OR infant:ti,ab,kw OR paediatrics:ti,ab,kw) AND smartphone:ti,ab,kw OR ‘mobile phone’:ti,ab,kw OR tablet:ti,ab,kw OR ‘mobile device’:ti,ab,kw OR ‘screen device’:ti,ab,kw) AND language:ti,ab,kw AND ‘language development’:ti,ab,kw AND [2010–2024]/py
Scopus	(TITLE‐ABS‐KEY (children) OR TITLE‐ABS‐KEY (kids) OR TITLE‐ABS‐KEY (“early childhood”) OR TITLE‐ABS‐KEY (toddler) OR TITLE‐ABS‐KEY (preschooler) OR TITLE‐ABS‐KEY (child) OR TITLE‐ABS‐KEY (“child, preschool”) OR TITLE‐ABS‐KEY (infant) OR TITLE‐ABS‐KEY (paediatrics) AND TITLE‐ABS‐KEY (smartphone) OR TITLE‐ABS‐KEY (phone) OR TITLE‐ABS‐KEY (cell) OR TITLE‐ABS‐KEY (mobile) OR TITLE‐ABS‐KEY (tablets) OR TITLE‐ABS‐KEY (screens) OR TITLE‐ABS‐KEY (“interactive screens”) OR TITLE‐ABS‐KEY (“mobile device”) OR TITLE‐ABS‐KEY (“screen device”) AND TITLE‐ABS‐KEY (language) OR TITLE‐ABS‐KEY (“language development”)) AND PUBYEAR > 2009 AND PUBYEAR < 2024 AND (LIMIT‐TO (LANGUAGE, “English”) OR LIMIT‐TO (LANGUAGE, “Spanish”) OR LIMIT‐TO (LANGUAGE, “Portuguese”)) AND (LIMIT‐TO (DOCTYPE, “ar”))
Google Scholar	(child) OR (child, preschool) OR (infant) OR (paediatrics) AND (smartphone) OR (tablets) AND (language) OR (language development)

### 2.4 Eligibility Criteria

The establishment of eligibility criteria aimed to ensure the inclusion of studies evaluating the impact of smartphone and tablet use on language development in children. The inclusion criteria were articles employing a cross‐sectional, longitudinal, or case–control design; studies involving children aged 0–6 years; studies assessing the impact of smartphone and tablet use on language development; original articles written in Portuguese, English or Spanish; and published between 2010 and 2024.

The exclusion criteria were as follows: (1) studies including children with congenital or acquired diseases that could interfere with language development; (2) intervention studies, as the PECOS framework focused on exposure rather than intervention; (3) studies that, when evaluating exposure to different types of screens, did not specify results related to the use of smartphones and tablets; and (4) publications classified as editorials, letters to the editor, or similar.

For the purposes of this review, language development was defined as encompassing both receptive and expressive domains. Studies assessing broader communication skills (e.g., vocabulary and interactional abilities) were also included. Eligible measures comprised standardized assessments and parent‐report questionnaires. Communication skills were understood as broader competencies beyond receptive and expressive language, including vocabulary, lexical density, sentence use, gestural‐mimetic abilities and early literacy skills, as defined in the included studies.

Interactive screen use was defined as the use of tablets or smartphones. Both active use (e.g., apps, educational games and interactive activities) and passive use (e.g., video watching on these devices) were considered. Studies assessing only television or computer use were excluded. Information on frequency or duration of use was extracted when reported.

### 2.5 Selection of Articles

**FIGURE 1:**
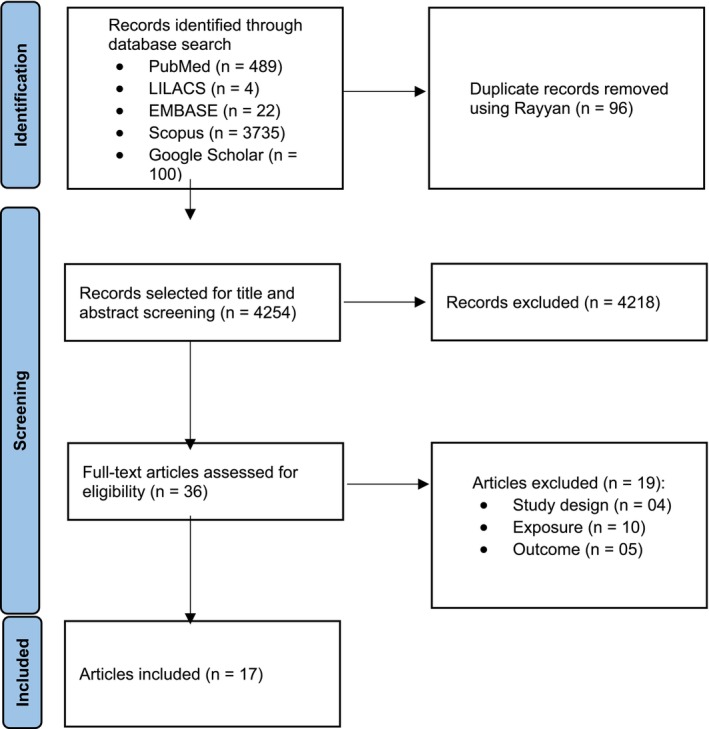
Study selection flowchart.

The searches, according to the strategies that had been previously defined, were carried out in December of 2023 and subsequently updated in May of 2024. The titles and abstracts obtained from the electronic search were selected independently by two authors (DCSJ and VB). Disagreements were resolved by consensus between the two reviewers. The relevant studies were thoroughly reviewed in their entirety and selected according to the predetermined eligibility criteria. The study selection process was documented using a flowchart in accordance with the PRISMA statement, as illustrated in Figure 1. (Page et al. 2021). The search results were imported into the Rayyan software, where duplicates were automatically detected and removed, thereby optimizing the process of screening eligible studies.

### 2.6 Data Extraction

The relevant information from all the included studies was extracted independently by two authors (DCSJ and VB) using a standardized spreadsheet. The following information was extracted: authors, year, country, journal, population, study design, outcomes and main results.

### 2.7 Assessing the Risk of Bias

The risk of bias assessment was conducted using the Joanna Briggs Institute (JBI) Risk of Bias Assessment Tool, available at https://jbi.global/critical‐appraisal‐tools. The selection of this instrument was driven by two key factors. Firstly, it has gained widespread acceptance within the research community due to its methodological rigor when evaluating the quality of the included studies. Following the selection of the relevant studies for the systematic review, two independent reviewers (DCSJ and VB) individually assessed the risk of bias of each study, using both the JBI tool for cross‐sectional studies and for case–control and cohort studies. These tools include a list of specific criteria for observational studies. The tool's items were evaluated to ascertain their potential for high, low, or uncertain risk of bias. Any discrepancies in the evaluation of risk of bias were addressed through deliberations among the reviewers or, when required, with the involvement of a third reviewer, with the objective of achieving a consensus.

## 3 Results

### 3.1 Study Selection

The preliminary search identified 4350 references, of which 489 were collected from PubMed, 22 from EMBASE, 4 from LILACS, 3735 from Scopus and 100 from Google Scholar. Following a thorough evaluation of the titles and abstracts, 36 full‐text articles were subjected to a comprehensive eligibility assessment, resulting in the exclusion of 19 articles. The initial selection was reduced by four studies due to the study design and by a further 10 studies that did not specify results related to the use of smartphones and tablets or that assessed other means of screen exposure. In addition, five studies were excluded on the basis of the absence of the outcome of interest (see Table 2). Following a comprehensive review of the extant literature, a total of 17 studies were included in the final analysis.

**TABLE 2:** Excluded studies and reasons for exclusions.

Author, year	Título do Article title	DOI	Reason
Zack, 2013	15‐month‐olds' transfer of learning between touch screen and real‐world displays: language cues and cognitive loads	10.1111/sjop.12001	Design: Intervention study
Alam, 2017	The impact of mobile phone‐based messages on maternal and child healthcare behaviour: a retrospective cross‐sectional survey in Bangladesh	10.1186/s12913‐017‐2361‐6	Outcome: Does not evaluate language
Farah, 2019	Hyperconnectivity during screen‐based stories listening is associated with lower narrative comprehension in preschool children exposed to screens vs. dialogic reading: An EEG study.	10.1371/journal.pone.0225445	Design: Intervention study
Martinot, 2021	Exposure to screens and children's language development in the EDEN mother–child cohort	10.1038/s41598‐021‐90 867‐3	Exposure: TV
Tomopoulos, 2010	Infant Media Exposure and Toddler Development	10.1001/archpediatrics.2010.235	Exposure: TV
Rosenqvist, 2016	Relationship of TV watching, computer use, and reading to children's neurocognitive functions	10.1016/j.appdev.2016.04.006	Exposure: TV and computer
McKean, 2015	Levers for Language Growth: Characteristics and Predictors of Language Trajectories between 4 and 7 Years	10.1371/journal.pone.0134251	Exposure: TV
Duch, 2013	Association of screen time use and language development in Hispanic toddlers: a cross‐sectional and longitudinal study	10.1177/0009922813492881	Exposure: The results have not been described separately for the use of smartphones or tables
Castles, 2013	Computer use and letter knowledge in pre‐school children: a population‐based study	10.1111/jpc.12126	Exposure: TV and computer
Wu, 2023	Effects of mobile device use on emotional and behavioural problems in the CBCL among preschoolers: Do shared reading and maternal depression matter?	10.1371/journal.pone.0280319	Outcome: Does not evaluate language
Morris, 2022	The impact of parents' smartphone use on language development in young children	10.1111/cdep.12449	Design: narrative review
Neumann, 2020	The Impact of Tablets and Apps on Language Development	10.1080/00094056.2020.1846394	Design: narrative review
Martzog & Suggate, 2022	Screen media are associated with fine motor skill development in preschool children	10.1016/j.ecresq.2022.03.010	Outcome: Does not evaluate language
McArthur, 2022	Screen time and developmental and behavioural outcomes for preschool children	10.1038/s41390‐021‐01572‐w	Exposure: TV and computer
Takahashi, 2023	Screen Time at Age 1 Year and Communication and Problem‐Solving Developmental Delay at 2 and 4 Years	10.1001/jamapediatrics.2023.3057	Exposure: The results have not been described separately for the use of smartphones or tables
Kerai, 2022	Screen time and developmental health: results from an early childhood study in Canada	10.1186/s12889‐022‐12 701‐3	Exposure: The results have not been described separately for the use of smartphones or tables
Veraksa, 2021	Short‐ and Long‐Term Effects of Passive and Active Screen Time on Young Children's Phonological Memory	10.3389/feduc.2021.600687	Exposure: The results have not been described separately for the use of smartphones or tables
Park, 2021	Smartphone use patterns and problematic smartphone use among preschool children	10.1371/journal.pone.0244276	Outcome: Does not evaluate language
Chang, 2018	Electronic Media Exposure and Use among Toddlers	10.30773/pi.2017.11.30.2	Outcome: Does not evaluate language

### 3.2 The Characteristics of the Included Studies

The studies selected for this systematic review addressed the impact of the use of interactive digital devices (smartphones and tablets) by preschool children on language and communication skills. Although some articles originally used broader terms such as “electronic devices” or “media exposure,” in this review, we report their findings specifically when referring to smartphones and/or tablets. The majority of studies were conducted between 2015 and 2024 and were undertaken in various countries. The majority of studies employed a cross‐sectional design, while one study was defined as a case–control study. The populations studied vary in terms of age, ranging from 6 months to 6 years. The outcomes assessed include, but are not limited to, language delays, literacy skills, gestural–mimetic communication skills and lexical density.

The studies included in this systematic review used a variety of instruments to measure the outcomes of interest related to language development and communication skills in preschool children. Among the most common instruments are standardized scales, such as the *Bayley Scales of Infant and Toddler Development, Clinical Evaluation of Language Fundamentals, Communicative Development Inventory*, among others. In addition, some studies have used interviews with parents, clinical assessments and specific questionnaires to identify language delays, literacy skills, gestural‐mimetic communication skills and lexical density (Table 3).

**TABLE 3:** Information extracted from the included studies.

First author	Year/country/periodical	Population	Study design	Exhibition (use of interactive screens)	Outcomes (instruments and measures of effect)	Main results	Limitations of the studies
Slobodin	2024/ Israel/Infancy	179 children aged 24 to 36 months	Cross‐sectional study	Screen use was assessed at 6, 12 and 24 months via online parental questionnaires. Exposure was 34.5% (6 months), 56.5% (12 months) and 94.5% (24 months). At 24 months, 13% of children used screens for more than 2 h a day.	Language and communication delays were analysed by medical records and referral history. Regression models examined the relationship between screen time and language deficits at 24 and 36 months, adjusting for factors such as socioeconomic status and gender.	Language delays were observed in 33% of children at 24 months and 15.1% at 36 months. Early exposure to screens (6 months) showed an association with language deficits at 36 months, especially in families of moderate to high socioeconomic status (𝛽 = 0.64; EP = 0.19; *p* < 0.001).	The study's limitations include bias in parental reports, a sample restricted to an Israeli health service, a high dropout rate and possible interference from the COVID‐19 pandemic in the results.
Chowsomchat	2023/Thailand/BMC Paediatrics	317 children aged 5 to 6	Cross‐sectional study	Screen use was assessed using a parental questionnaire, considering interaction with cell phones, tablets, TVs, computers and video games. The prevalence was 79.5%, but the study did not detail the length of exposure.	Literacy skills were assessed with Rama Pre‐Read (RPR) software. Logistic regression models used OR and 95% CI to analyse the relationship between the use of touchscreen devices and the development of emergent literacy.	The study did not provide data on the prevalence of language delays. There was no association between the use of touchscreen devices and phonological awareness, automated rapid naming or letter naming (*p* > 0.05).	Limitations of the study include the restricted assessment of phonological awareness and rapid naming, possible self‐report bias in the measurement of screen use and the geographical limitation to the Thai context, making it difficult to generalize the results.
Turco	2023/EUA/British Journal of Educational Technology	881 children aged 4 to 5	Cross‐sectional study	Screen use was assessed by parental online questionnaires, reporting frequency and daily duration. On average, children used mobile devices for 49 min a day.	Letter/word recognition (Woodcock Johnson‐III), phonological awareness (PALS‐PreK) and language (QUILS) were assessed. The relationship between the use of mobile devices and language development and emergent literacy was analysed by multiple regression.	The study did not present data on the prevalence of language delays. The regressions indicated that the use of mobile devices had no significant association with language and literacy, explaining minimal variation: each increase in usage time reduced language processing and vocabulary skills by 0.06 points (*p* < 0.001).	Limitations of the study include the reliance on parental reports, which are susceptible to self‐report bias, and the absence of data on the content and quality of interaction with devices, essential factors for assessing their impact on language and literacy development.
Al Hosani	2023/United Arab Emirates/Middle East Current Psychiatry	227 children with language delay and 227 normal children aged 12 to 48 months	Case–control	Screen use was assessed by questionnaires on the age at which exposure began and the daily time spent on television and electronic devices. The study revealed that 88.3% of children were exposed to screens before the age of 24 months.	Speech and language delays were assessed by interviews with parents and the RELT test. The association between the use of screens and these delays was analysed using OR with 95% CI.	The study revealed that 90.3% of children with language delay used electronic devices. Early use (12–24 months) significantly increased the risk of delays (OR = 6.82; *p* < 0.001), while late use (25–36 months) had a positive effect on language development (OR = 0.32; *p* = 0.017).	The main limitations are the possibility of recall bias, as well as the lack of measures to assess important variables such as interactive activity and parenting style.
Gath	2023/New Zealand/Current Research in Behavioral Sciences	84 children aged 3 and 5	Cross‐sectional study	Screen use was assessed using an online questionnaire completed by parents. On average, children spent 1.78 h a day on weekdays and 2.65 h on weekends.	The relationship between screen time and language skills was assessed by Pearson correlations (r), using CELF‐P2 for oral production and a story retelling task for listening comprehension.	The use of tablets and cell phones showed a negative correlation with children's language skills (−0.27 for production and −0.22 for comprehension). However, the association with comprehension was not statistically significant (*p* = 0.08).	The study's limitations include its correlational design, which prevents causal inferences, the reliance on parental reports of screen time and the lack of analysis of co‐viewing and types of use, elements that could shed more light on the impacts on language development and the bond between parents and children.
Gago‐Galvagno	2023/Argentina/Trends in Psychology	114 of children aged 12 and 36 months	Cross‐sectional study	The use of tablets and cell phones by children was assessed using questionnaires, in which parents reported the daily duration of use of these devices. On average, children used tablets for around 1.34 min and cell phones for around 32.93 min a day.	Lexical density and the use of sentences (Communicative Development Inventory). The effect measures used to analyse the impact of screen use on language development included Spearman correlation coefficients (rho) and hierarchical linear regressions.	The study did not provide data on the overall prevalence of language delays. Cell phone use was negatively correlated with lexical density (*rho* = −0.23), while tablet use had a positive association with sentence construction (*rho* = 0.25). However, in the linear regression, these associations lost significance after adjusting for socioeconomic and demographic variables.	The main limitations of the study include the use of parental reports which can introduce self‐report bias, the lack of control over the content and quality of the interaction with the screens, and the cross‐sectional nature of the study which limits the inference of causality.
Jamil	2023/Iraq/Advanced Medical Journal	300 children aged 12 to 36 months	Cross‐sectional study	The use of smartphones by children was assessed using questionnaires that recorded the hours of daily use and the content of the videos watched (age‐appropriate or not). The prevalence of screen use among the children assessed was 80%.	The study used the CDC's developmental milestones scale to assess speech and language delays. Chi‐square tests were used to analyse the association between smartphone use and language acquisition, determining the statistical significance of the relationships observed.	The study showed that 58% of the children showed language delay. Among smartphone users, the rate was 59.6%, compared to 51.7% of non‐users (*p* = 0.266). The delay was more prevalent in children who used smartphones for 4 h or more a day, reaching 76.1% (*p* = 0.003).	The main limitations of the study include its cross‐sectional design, which limits the ability to infer causality, and the reliance on self‐administered questionnaires, which can introduce self‐reporting bias.
Chaibal & Chaiyakul	2022/Thailand/Acta Psychologica	85 children between 2 and 5 years old	Cross‐sectional study	Screen use by children was assessed through interviews with the main caregivers, who reported their daily use of smartphones and tablets over seven consecutive days. The average age of onset of screen use was 2.77 years and the average time of use reported was 82.78 min per day.	Language development (Denver Developmental Screening Test II). The effect measures used to assess the impact of smartphone and tablet use on children's development included Pearson correlations (r) and chi‐square tests.	The study did not identify language delays as a direct result. The length of time children used smartphones and tablets was positively correlated with maternal use (*r* = 0.294, *p* = 0.015) and parental use (*r* = 0.703, *p* = 0.002), with no significant association with language development (*p* = 0.614).	The main limitations of the study include its cross‐sectional design, which prevents the inference of causality, the small sample size and the reliance on caregivers' reports, which can introduce self‐reporting bias.
Schwarzer	2022/Germany/Paediatric Research	296 children between 2 and 5 years old	Cross‐sectional study	Screen use was assessed by parental questionnaires, recording the average daily time spent on TV, consoles, computers/tablets and cell phones. The total average time was 0.75 h/day, with 24% of children exceeding the recommendation (> 1 h/day).	Language skills were assessed using the Development Test 6 Months to 6 years—Revision (ET 6–6‐R). The associations between screen time and child development (cognitive, linguistic and socio‐emotional) were analysed using linear regression coefficients.	The study did not report the specific prevalence of language delays, but identified that high screen times (> 1 h per day) were significantly associated with lower scores in language skills, with a regression coefficient of 𝛽 = −12.88 (95% CI −20.19 to −5.57, *p* < 0.01).	The main limitations include the use of parental questionnaires, which are subject to self‐report bias, the lack of evaluation of the specific content consumed on screens, and the cross‐sectional nature of the study, which limits the inference of causality.
Dynia	2021/EUA/Infant Behavior and Development	157 children between 33 and 36 months	Cross‐sectional study	Media exposure was assessed through mothers' self‐reports on the amount of time their children spent in front of screens. The average media exposure was 3.79 h per day, with only 24% of children meeting the American Paediatric Association's recommendation of 1 h or less per day.	Language skills were assessed using the Bayley Scales and the Peabody Picture Vocabulary Test. The association with media exposure was analysed by linear regression, measuring impacts on expressive and receptive language and receptive vocabulary.	The study did not provide data on the prevalence of language delays. Media exposure was negatively associated with expressive language ( = − 0.29; *p* = 0.01), but there was no significant association with receptive language or vocabulary.	The main limitations include the use of parental self‐reports, which can introduce memory bias and social desirability, and the lack of evaluation of the content and context of the media consumed, limiting the understanding of the specific impacts on language.
Salunkhe	2021/Indía/Medical Journal of Dr. D.Y. Patil Vidyapeeth	435 children aged 6 months to 6 years	Cross‐sectional study	The use of electronic media was assessed through parents' reports on how much time children spent using these devices each day. The study found that 75.2% of children aged between 3 and 6 used smartphones, and 55.81% of children under 3 did the same.	Speech delay (Language Evaluation Scale Trivandrum [LEST]). The effect measures used to assess the association between the use of electronic media and language delay were not specified.	Language delay was observed in 28.4% of children with more than 3 h of media daily, compared to 23.4% among non‐users, indicating a significant association between prolonged screen use and language delay.	The main limitations include the reliance on parental self‐reports, which are susceptible to memory bias, and the lack of control over the content and context of electronic media use, limiting the generalizability of the results.
Operto	2020/Italy/Brain Sciences	260 children aged 8 to 36 months	Cross‐sectional study	The study assessed the use of digital devices by means of parental questionnaires, recording age of onset, daily time and type of device. Use was reported by 97% of the children, with an average of 2.13 h/day, with smartphones being the most used (66%).	The study evaluated the relationship between the use of digital devices and gestural and lexical communication skills by means of linear regression analysis, using the “Il primo vocabolario del bambino PVB” (“Gesti e Parole” and “Parole e Frasi” forms).	The study revealed a negative association between the time spent using digital devices and language skills. In children aged 8 to 17 months, it affected actions and gestures (𝛽 = − 0.397; *p* = 0.001), while in children aged 18 to 36 months, it impacted the lexical quotient ( = − 0.224; *p* = 0.001).	Limitations include the use of self‐reported questionnaires by parents, subject to memory bias, and the lack of distinction between the types of content accessed, making it difficult to assess specific impacts on language development.
Nobre	2020/Brazil/Jornal de Pediatria	104 children aged 23 to 42 months	Cross‐sectional study	The study assessed the use of interactive media by parental questionnaires. The median time of daily use was 45 min, ranging up to 8 h. Smartphones were used by 88.5% of the children, while 37.5% used tablets.	The study used Spearman correlations and linear regression to assess the relationship between interactive media and child development, including language, cognition and motor skills.	The study did not directly measure the prevalence of language delays. However, it did find a positive correlation between the quality of interactive media use and language development (*rho* = 0.40; *p* < 0.001), explaining 20% of the variation (*r* ^2^ = 0.20; *p* < 0.001).	The main limitations include the use of self‐reported questionnaires by parents, subject to recall bias, and the cross‐sectional nature of the study, which prevents the inference of causality between the use of interactive media and child development. Children.
Moon	2019/South Korea/Acta Paediatrica, International Journal of Paediatrics	117 children aged 3 to 5	Cross‐sectional study	The use of smart devices by children aged three to five was assessed by parental questionnaires. The majority used them between one and four times a week (67.5%) and for less than an hour a day (60.7% at weekends and 70.1% on weekdays).	Language development (Preschool Receptive‐Expressive Scale). The effect measures used to assess the relationship between the use of smart devices and language development included Spearman correlations (rho).	The study did not directly measure the prevalence of language delays. However, it did find a significant negative correlation between the time of device use and the age of expressive language in three‐year‐olds (*rho* = −0.481; *p* < 0.01).	The main limitations include the cross‐sectional design, which prevents causal inferences, and the reliance on parental self‐reports, which can introduce memory and social desirability bias.
Van den Heuvel	2018/Canada/Journal of Developmental & Behavioral	893 children aged 18 months	Cross‐sectional study	The use of mobile devices by 18‐month‐old children was assessed through a questionnaire completed by parents, who reported the average daily time spent using devices such as smartphones and tablets. The majority of parents (77.6%) reported that their children did not use mobile devices, while 22.4% of children used these devices, with a median of 15.7 min per day.	Speech delay and other communication delays (Infant Toddler Checklist). The effect measures used included odds ratios (OR) in logistic regression models to assess the association between the use of mobile devices and delays in expressive language.	The study reported that 6.6% of the children had expressive language delay and 8.8% had other communication delays. Each 30‐min increase in daily mobile device use significantly increased the odds of expressive speech delay (adjusted OR = 2.33; 95% CI: 1.25–4.82; *p* < 0.01).	The main limitations of the study include the use of parental self‐reports, which are susceptible to recall bias, and the lack of information on the content and context of mobile device use, as well as the cross‐sectional design, which prevents causal inference.
Taylor	2017/UK/Journal of Children and Media	131 children between 6 and 36 months	Cross‐sectional study	The study assessed the use of screen media by children aged between 6 and 36 months via an online questionnaire completed by their parents. The results showed that 49% of children used mobile devices on a daily basis, with the average time varying according to age and the type of media used.	The study used regression analyses to assess the relationship between screen media exposure time and the size of children's expressive and receptive vocabulary, based on the Communicative Development Inventory.	The study did not directly measure the prevalence of language delays. Furthermore, it found no significant association between TV or mobile device use and vocabulary size, after adjusting for factors such as reading time and non‐media activities.	The main limitations of the study include the homogeneous sample of highly educated families, which limits the generalizability of the results, and the use of parental self‐reports, which may introduce recall bias.
Rajchanovska & Ivanovska	2015/Macedonia/Srp Arh Celok Lek	1607 children between 3 and 5 years old	Cross‐sectional study	The study assessed the use of electronic devices by children through interviews with parents. The prevalence of cell phone use was 24.6%, but the average daily time of use was not specified in detail.	The study assessed speech disorders through interviews with parents, clinical examination and the Child Behaviour Checklist questionnaire. Logistic regression and chi‐square tests were used to analyse the relationship between the use of electronic devices and speech disorders in children.	The prevalence of speech disorders was 37.7%. An association was observed between cell phone use and speech disorders (OR = 1.389; 95% CI 1.133–1.702, *p* = 0.002).	The main limitations of the study are the use of parental self‐reports, susceptible to recall and interpretation bias, and the lack of control over the content and context of the use of electronic devices, which limits the generalization of the results.

Abbreviations: CI, confidence interval; OR, odds ratio; SE, standard error.

Across the included studies, exposure to smartphones and tablets was measured in different ways: dichotomously (e.g., user vs. non‐user), continuously (e.g., average daily use in hours) or categorically (e.g., < 30 min, 1–2 h and > 2 h per day). When reporting the results, this review specifies how exposure was operationalized in each study.

### 3.3 Results of Individual Studies

An in‐depth analysis of the main findings of the studies included in this systematic review reveals a complex relationship between preschool children's use of digital devices and their development of language and communication skills (Table 4). On the one hand, studies such as those by Al Hosani et al. (2023), Slobodin et al. (2024) and Gath et al. (2023) found worrying links between heavy use of electronic devices, such as mobile phones and tablets, and delays in language development in preschool children.

**TABLE 4:** Impact of mobile devices (tablets and smartphones) on children's language.

First author	Negative impact	Positive impact	No evidence of impact
Slobodin	🙁		
Chowsomchat			😑
Turco			😑
Al Hosani	🙁		
Gath	🙁		
Gago‐Galvagno	🙁	☺	
Jamil	🙁		
Chaibal and Chaiyakul			😑
Schwarzer			😑
Dynia	🙁		
Salunkhe	🙁		
Operto	🙁		
Nobre		☺	
Moon	🙁		
van den Heuvel	🙁		
Taylor			😑
Rajchanovska and Ivanovska	🙁		

*Note:* 🙁(Negative impact) ☺(Positive impact) 😑 (No evidence of impact).

On the other hand, there is evidence to suggest that the use of digital devices may be beneficial for children's language. Studies such as Gago‐Galvagno et al. (2023) report that mobile phone use was negatively associated with lexical density and sentence use, whereas tablet use and shared TV viewing with adults were positively associated with increased lexical density and sentence use in children. These findings highlight the complexity of the relationship between technology use and language development, suggesting that the context of use and the specific type of device may play an important role in the observed results.

However, some of the studies reviewed found no significant associations between digital device use and language development in preschool children. Studies such as Turco et al. (2023) found no significant effects of the use of mobile digital devices on language skills after adjusting for demographic characteristics and family income. This lack of direct association highlights the complexity of the issue and the need for further research to fully understand the impact of digital devices in early childhood, taking into account not only quantitative aspects but also the context and quality of use of these devices (Table 4).

### 3.4 Summary of Results

The results of the studies analyzed on the association between the use of electronic devices and language delays in children are markedly heterogeneous, reflecting differences in methodology, populations studied and contexts of use. The studies that found a significant association between the use of electronic devices and language delays present a worrying scenario regarding the impact of early and prolonged exposure to these technologies on children's development. The study conducted by Al Hosani et al. (2023) in the United Arab Emirates found that the vast majority of children with speech and language delays (90.3%) used electronic devices and were almost seven times more likely to have these delays than children who did not use such devices (OR = 6.82; 95% CI: 4.09–11.40; *p* < 0.001). Similarly, Van Den Heuvel et al. (2019), in a Canadian study, showed that each 30‐min increase in daily use of mobile devices among 18‐month‐old children was associated with a doubling of the odds of delay in expressive speech (adjusted OR of 2.33; 95% CI: 1.25–4.82; *p* < 0.001). Rajchanovska and Ivanovska (2015), in a large study in Macedonia, also reported a significant association between cell phone use and speech disorders, with 38.9% increased odds of such disorders among cell phone users (OR = 1.389; 95% CI: 1.133–1.702; *p* = 0.002).

In addition, some studies have shown negative associations, although not directly related to severe language delay. Schwarzer et al. (2022), in a German study, found that high screen time, defined as more than 1 h per day, was significantly associated with lower language skills, with a regression coefficient of −12.88 (95% CI: −20.19 to −5.57; *p* < 0.01). Similarly, Dynia et al. (2021), in a North American study, demonstrated that media exposure was negatively associated with children's expressive language skills, with a regression coefficient of −0.29 (*p* = 0.01). These findings suggest that, while electronic media use is not universally associated with severe language delays, its adverse impact on the quality and development of language skills is evident under specific exposure conditions.

Conversely, there are studies that do not corroborate this direct relationship between the use of devices and language delays. Chowsomchat et al. (2023), in a study of Thai children, revealed no statistically significant correlation between touchscreen usage and emergent literacy skills. In a similar vein, Turco et al. (2023) in a North American study, concluded that the use of mobile devices by young children was not significantly associated with the development of language and literacy skills. However, the study found a slight decrease in language processing skills and vocabulary with increasing screen time. These findings suggest that the relationship between screen time and language development may be more complex and influenced by other contextual factors.

Finally, there are studies that have not observed significant impacts of the use of electronic devices on language development. Taylor et al. (2018), in a study conducted in the United Kingdom, found no significant association between mobile device use and children's vocabulary size, even after adjusting for factors such as reading time and non‐media activities. Similarly, Gago‐Galvagno et al. (2023), in a study conducted in Argentina, revealed no substantial associations between the utilization of tablets and cell phones and language development. This observation was made after controlling for socioeconomic and demographic variables. However, the study did identify preliminary correlations that suggested potential differential effects on sentence use and lexical density. The extant literature suggests that the impact of device use may be mitigated by contextual and individual factors, such as the family environment and the quality of interactions during screen time.

### 3.5 Risk of Bias Assessment

Some studies presented areas of uncertainty or specific methodological deficiencies. Chowsomchat et al. (2023) and Schwarzer et al. (2022) were not specific in measuring exposure and in identifying and addressing confounding factors (Table 5). Turco et al. (2023) demonstrated a lack of clarity across several criteria, including the measurement of exposure and the identification of confounding factors. Jamil et al. (2023) and Salunkhe et al. (2021) had gaps in the confounding factors and the strategy to deal with them. Nobre et al. (2020) did not clearly define the inclusion criteria and due to inconsistencies in the identification and treatment of confounding factors. These points of attention indicate areas for methodological improvement in future studies to ensure greater rigour and minimize the risk of bias.

**TABLE 5:** Assessment of the risk of bias of the included cross‐sectional studies.

First author	Q1	Q2	Q3	Q4	Q5	Q6	Q7	Q8
Slobodin	Y	Y	Y	Y	Y	Y	Y	Y
Chowsomchat	Y	Y	UN	UN	Y	UN	UN	Y
Turco	Y	Y	UN	UN	UN	UN	UN	UN
Gath	Y	Y	Y	Y	Y	Y	Y	Y
Gago‐Galvagno	Y	Y	Y	Y	Y	Y	Y	Y
Jamil	Y	Y	UN	Y	Y	N	Y	Y
Chaibal and Chaiyakul	Y	Y	Y	Y	Y	Y	Y	Y
Schwarzer	Y	Y	UN	Y	Y	Y	Y	Y
Dynia	Y	Y	UN	Y	Y	Y	Y	Y
Salunkhe	Y	Y	Y	Y	Y	N	Y	Y
Operto	Y	Y	Y	Y	Y	Y	Y	Y
Nobre	N	N	UN	Y	N	N	Y	Y
Moon	Y	Y	Y	Y	Y	Y	Y	Y
van den Heuvel	Y	Y	Y	Y	Y	Y	Y	Y
Taylor	Y	Y	Y	Y	Y	Y	Y	Y
Rajchanovska and Ivanovska	Y	Y	Y	Y	Y	Y	Y	Y

*Note:* Y: yes; N: no; NA: not applicable; UN: unclear. (1) Comparability of groups; (2) Proper pairing; (3) Criteria for identifying cases and controls; (4) Exposure measurement; (5) Consistency in measuring exposure; (6) Identification of confounding factors; (7) Strategies for dealing with confounding factors; (8) Assessment of outcomes.

The study Al Hosani et al. (2023) was assessed for risk of bias following the Joanna Briggs Institute guidelines, and the results indicate that it obtained a positive answer (“Y”) for all questions considered. The groups were comparable except for the presence or absence of disease; cases and controls were appropriately matched, and the same criteria were used to identify the groups. Exposure was measured in a standardized, valid and reliable manner, and the same method was used for both cases and controls. In addition, confounding factors were identified, and strategies for dealing with them were demonstrated. Outcome assessments were performed in a standardized, valid, and reliable manner for both groups. The exposure period was sufficiently long to be meaningful, and appropriate statistical analysis was used. These positive responses suggest that the study has a low risk of bias (Table 6).

**TABLE 6:** Assessment of the risk of bias of the included case–control study.

First author	Q1	Q2	Q3	Q4	Q5	Q6	Q7	Q8	Q9	Q10
Al Hosani	Y	Y	Y	Y	Y	Y	Y	Y	Y	Y

*Note:* Y: yes; N: no; NA: not applicable; UN: unclear. (1) Comparability of groups; (2) Proper pairing; (3) Criteria for identifying cases and controls; (4) Exposure measurement; (5) Consistency in measuring exposure; (6) Identification of confounding factors; (7) Strategies for dealing with confounding factors; (8) Assessment of outcomes; (9) Adequate exposure period; (10) Appropriate statistical analysis.

The synthesis of findings was conducted narratively. While no formal weighting scheme was applied, the assessment of the risk of bias guided the interpretation of results. Greater emphasis was placed on studies with a lower risk of bias, whereas findings from studies with methodological limitations were interpreted more cautiously.

## 4 Discussion

The present systematic review sought to analyse the impact of interactive screens on language development in children up to 6 years of age. This topic has generated significant discussions due to the potential positive and negative effects of early exposure to technology. A review of the extant literature reveals that, while exposure to screens may offer certain benefits in specific circumstances, it is also associated with risks for language development, particularly in contexts of excessive use and without parental supervision.

These findings are consistent with previous systematic reviews, such as Madigan, McArthur, et al. (2020) who also reported that while a greater quantity of screen time tends to correlate with poorer language skills, aspects like co‐viewing and educational content can mitigate or reverse these associations. Similarly, Massaroni et al. (2023) found that during the first 2 years of life, screen exposure, especially passively, was particularly harmful for vocabulary and comprehension. These findings align with ours in terms of negative associations and also highlight the moderating role of content and supervision.

The findings of the review show that some research, such as that of Al Hosani et al. (2023) and Gath et al. (2023), have identified a negative correlation between screen time and language development. The extant literature suggests that prolonged exposure to digital devices may have a deleterious effect on the acquisition of fundamental language skills, particularly in children at an early developmental age. The findings of this study are of concern, as language development at this particular stage is imperative for the establishment of communicative and cognitive skills. These skills are essential for long‐term learning and social development (Yang et al. 2024).

Conversely, alternative perspectives have been proposed by Gago‐Galvagno et al. (2023), highlighting the potential benefits of digital device utilization, particularly in adult content sharing contexts, including the promotion of lexical density and complex sentence structures. These results are consistent with the findings of a recent study, which indicates that co‐viewing content, defined as parents or caregivers interacting and discussing the material watched with the child, can effectively enhance vocabulary and stimulate linguistic development (Sundqvist et al. 2022). These findings underscore the notion that the effects of screen use are not uniform and are contingent on factors such as the type of content consumed, the quality of interaction and parental support during technology use.

The heterogeneity of the results obtained in this review indicates that the effect of screen time on language development is malleable. This underscores the necessity of contemplating the quality and nature of digital content, as well as the supervision and social interaction during its utilization. Pedrotti et al. (2024) underscore the necessity of incorporating screen time into analyses and taking into account contextual variables, as well as the role of the family environment in mediating technology use.

Among the studies that did not find significant associations between screen use and language development, the study by Turco et al. (2023) is particularly noteworthy. These results suggest that other factors, such as socioeconomic characteristics, access to educational resources and family interactions, play a more significant role in language acquisition than technology use alone. This conclusion is corroborated by research showing that the quality of parental interactions, the presence of reading activities and varied cognitive stimulation are stronger predictors of language development than screen time (Madigan, McArthur, et al. 2020).

The review identified methodological limitations in several studies, including the lack of standardization in defining inclusion criteria, the absence of control over confounding factors and the limitation of cross‐sectional designs, which preclude causal inferences. The incorporation of longitudinal studies is imperative to enhance our comprehension of the long‐term ramifications of screen utilization during childhood. Furthermore, it is imperative to consider the nature of the content accessed, as exposure to educational and interactive content may have different effects compared to passive media consumption. Research conducted by Kumar Muppalla et al. (2023) and Nobre et al. (2020) underscores the efficacy of interactive and educational content, when coupled with social interaction, in promoting linguistic development. This stands in contrast to one‐dimensional or passive content. The quality of the content, in conjunction with the exposure duration and the incorporation of adult mediators, constitutes an element that merits consideration to facilitate a more comprehensive evaluation of the impacts of screen use.

This review contributes to the literature by specifically focusing on interactive screen use (such as tablets and smartphones) rather than treating all screen time as a homogeneous exposure. Synthesizing evidence across multiple contexts provides new insights into how factors such as co‐viewing, content type and parental mediation modulate language development outcomes. Unlike previous reviews, which largely examined television or undifferentiated screen time, this review isolates the effects of interactive devices, thereby offering more precise recommendations for parents, educators and policymakers.

The review underscores the necessity for educational interventions and public policies that promote the deliberate use of technology during childhood. Parents and educators should be encouraged to play an active role in their children's use of digital devices, ensuring that screen time is utilized productively and contributes to learning and language development. Collaboration between health professionals, educators and families is essential to create a balanced environment that maximizes the benefits of technology and minimizes the risks associated with excessive use.

It is imperative to underscore that the risk of bias was observed in select studies, including those by Chowsomchat et al. (2023) and Schwarzer et al. (2022). These studies failed to delineate strategies to control confounding factors, such as socioeconomic characteristics and the quality of the family environment. The absence of control over these variables may lead to the production of distorted conclusions and limit the applicability of the findings. To overcome these limitations, a more rigorous approach to the identification and control of confounding variables is recommended in future studies.

It is imperative to acknowledge the limitations of this review, which, despite its efforts to minimize bias and conduct a comprehensive search, is subject to certain restrictions. These limitations include the restriction of included studies to specific databases and the possibility of publication bias. Furthermore, the heterogeneity between the methods and populations studied may impact the generalizability of the results. Consequently, future research should persist in exploring this topic, employing more robust methodological approaches that consider the complexity of the factors that influence language development in children in a context of interactive screen use.

## 5 Conclusion

The systematic review demonstrated that the use of interactive screens in children up to 6 years of age can have negative effects and potential benefits, depending on the type of device, duration and context of use. While the majority of the studies exhibited a low to moderate risk of bias, areas that necessitate enhancement were identified, such as the standardization of exposure measurement and the control of confounding variables, to ensure greater methodological rigor in future research.

Therefore, it is essential that the use of interactive screens by preschool children be carefully monitored, prioritizing practices that encourage social interaction over isolated use. It is imperative that further research be conducted to enhance the comprehension of this relationship and furnish evidence‐based counsel for parents, educators and policymakers.

## Author Contributions

**Djalma Carmo da Silva Junior:** conceptualization, data curation, formal analysis, investigation, methodology, writing – original draft. **Yasmin Marques Castro:** data curation, formal analysis, writing – original draft. **Rinelly Pazinato Dutra:** investigation, methodology, writing – original draft. **Douglas Pinheiro Caumo:** investigation, methodology. **Michael Pereira da Silva:** formal analysis, investigation, methodology, project administration, supervision.

## Ethics Statement

Since this is a systematic review of previously published observational studies, no new data were collected from human participants. Therefore, ethical approval and informed consent were not required. All included studies were reviewed for appropriate ethical compliance based on their respective institutional approvals.

## Consent

The authors have nothing to report.

## Conflicts of Interest

The authors declare no conflicts of interest.

## Data Availability

The data analysed during this systematic review are publicly available through the original published studies. A complete list of all studies included in the review is available from the corresponding author upon request.
